# Adaptive laboratory evolution of *Saccharomyces cerevisiae* CEN.PK 113-7D to enhance ethanol tolerance

**DOI:** 10.1093/femsyr/foaf058

**Published:** 2025-11-28

**Authors:** Fatemeh Sheikhi, Mahsa Babaei, Khosrow Rostami, Mehrdad Azin, Mohammad Ali Asadollahi, Payam Ghiaci, Mansour Ebrahimi, Amir Feizi, Irina Borodina

**Affiliations:** Department of Biotechnology, Iranian Research Organization for Science and Technology (IROST), Tehran 331319-3685, Iran; Sugarcane Training and Research Institute, Khuzestan 63451-96316, Iran; The Novo Nordisk Foundation Center for Biosustainability, Technical University of Denmark, DK-2800 Kgs. Lyngby, Denmark; Department of Biotechnology, Iranian Research Organization for Science and Technology (IROST), Tehran 331319-3685, Iran; Department of Biotechnology, Iranian Research Organization for Science and Technology (IROST), Tehran 331319-3685, Iran; Department of Biotechnology, Faculty of Biological Science and Technology, University of Isfahan, Isfahan 81746-73441, Iran; Department of Biorefinery and Energy, High-throughput Centre, Research Institutes of Sweden, Örnsköldsvik 89250, Sweden; Bioinformatics Research Group, Green Research Center, University of Qom, Qom 3716133779, Iran; OMass Therapeutics, Oxford, OX4 2GX United Kingdom; The Novo Nordisk Foundation Center for Biosustainability, Technical University of Denmark, DK-2800 Kgs. Lyngby, Denmark

**Keywords:** Adaptive laboratory evolution, CRISPR-Cas9, ethanol tolerance, *Saccharomyces cerevisiae*, reverse engineering, strain improvement

## Abstract

*Saccharomyces cerevisiae* is a widely used yeast for industrial production of ethanol. However, elevated ethanol, temperature, and osmotic stress adversely affect fermentation efficiency. In this study, adaptive laboratory evolution for *S. cerevisiae* CEN.PK 113–7D on higher concentrations of ethanol was performed. After 144 days, the maximum specific growth rate (µ_max_) increased from 0.0240 to 0.1150 h^−1^ for the strain evolved on 9% v/v ethanol, and from 0.0002 to 0.0530 h^−1^ for the strain evolved on 11% v/v ethanol, and the specific glucose uptake rate increased by 30%. The strain evolved on 11% ethanol produced 94.5 g/L ethanol in a fermentation as compared to 78.5 g/L production by a non-evolved strain. By whole-genome sequencing of the evolved clones, we identified multiple coding mutations in genes involved in processes such as stress response, cell growth regulation, pentose phosphate pathway, lipid synthesis, and redox balance. The selected mutations *in RKI1, CYC2, ANR2, RGA2, RGA1, LPX1*, and *LRE1* genes were validated by introducing them in the nonevolved yeast, showing 1.7–5-fold growth improvement at 9% ethanol (*P* < 0.05). Notably, RGA2, RGA1 and LPX 1 carried an identical missense mutation across three independent clones. The RKI1^I208V^ mutant showed the highest ethanol tolerance, while CYC2^N342A^ achieved the highest ethanol production.

## Teaser

Several new mutations that increase ethanol tolerance in *Saccharomyces cerevisiae* were identified through adaptive laboratory evolution, followed by whole-genome resequencing and reverse engineering. Some of these mutations resulted in enhanced ethanol production. For the first time, novel genes associated with ethanol tolerance have been identified in this context.

Abbreviations ALE: Adaptive laboratory evolution
*S. cerevisiae*
: *Saccharomyces cerevisiae*

## Introduction

The budding yeast *Saccharomyces cerevisiae* has had a significant impact on human society, with a 9000-year history of application in baking, brewing, and distilling. Besides fermented beverages, ethanol, as a sustainable transportation fuel, has expanded the industrial use of budding yeast (Shou-Fu et al., 2018). In addition, the global use of alcohol-based hand sanitizers has risen significantly recently, as public health agencies worldwide have recommended hand hygiene as a preventive measure during the COVID-19 pandemic (Aboga and Nyamweya, 2021). Therefore, optimizing bioethanol production plays a key role in meeting the increasing demand for ethanol. Ethanol production in yeast has been improved by rational and evolutionary engineering. Evolutionary engineering is advantageous because the resulting strains are not considered GMO (Mans et al., 2018). Several studies have shown that an improvement in ethanol tolerance is associated with increased ethanol production (Stanley et al., 2010, Fiedurek et al., 2011, Mukherjee et al., 2014). Intracellular ethanol concentration affects cell growth more than extracellular ethanol, but the inhibitory effect of ethanol mainly depends on the balance between the ethanol production rate and its release outside the cell. Maintaining the cells' homeostasis conditions appropriately increases ethanol release (Dombek and Ingram, 1986, Mukherjee et al., 2014). Ethanol inhibits yeast growth and viability and generally affects the physiology of yeast cells (Arroyo Lopez et al., 2010, Auesukaree, 2017). High levels of ethanol impose stress on yeast cells through different mechanisms such as disruption of the cell membrane, damage to protein structures, dissipation of the transmembrane electrochemical potential, prevention of nutrient uptake by hexose transporters, reduction in the activity of the plasma membrane ATPase, and induction of water stress with a decrease in water activity (Bai et al., 2004, Teixeira et al., 2009, Auesukaree, 2017). The response of yeast cells to ethanol stress is complex, involving signal transduction, transcriptional and post-transcriptional control, and the enhanced activity of repair functions through small mutations in the genome (Stanley et al., 2010). Ethanol stress itself is a key factor that inhibits ethanol production in bioethanol fermentation processes. Consequently, there has been great interest in understanding the underlying mechanisms of ethanol toxicity and tolerance in *S. cerevisiae* strains. A large number of genes were identified as being implicated in ethanol tolerance (Table 1).

**Table 1.** *Saccharomyces cerevisiae* genes involved in ethanol tolerance

Gene	Function	Reference
*sod2*	Superoxide dismutase	(Costa et al., 1997)
*bem2*	Cytoskeleton organization and cellular morphogenesis	(Takahashi et al., 2001)
*pat1*	Topoisomerase II associated	
*rom2*	Expresses a GDP⁄GTP	
*vps34*	Synthesis of phosphatidylinositol 3-phosphate	
*ada2*	Transcriptional activator	
*pac10*	Cytoplasmic Gim proteins	(Fujita et al., 2006)
*gim4 and gim5*	N-terminal acetyl transferase	
*vma1, vma2, vma4, vma6, vma8, vma13* and *vma16*	ATP-dependent proton pump called vacuolar H^+^-ATPase (V-ATPase)	
*btn2*	Mediating pH homeostasis between the vacuole and plasma membrane	
*anc1, ard1, bfr1, bik1, bni1, bub1, cnm67, ctf4, elm1, grr1, hex3, hpr1, htl1, pol32, rad27, rsc2, shp1, shs1 ume6, vid21*	Cell cycle and DNA processing	
*alg6, doa4, gim4, gim5, lhs1, mft1, nat1, nat3, pac10, pfd1, ppm1, pre9, rad6, tom37, ump1, vps36, vps41, yme1, ynd1, yta7*	Protein fate	
*akr1, apn1, atp15, bro1, clc1, fen2, fps1, gtr1, isa2, luv1, she4, snf7, snf8, stp22, trs33, vps20, vps28*	Cellular transport mechanisms	
*caf16, cst6, ctk3, dhh1, elp2, elp6, iki3, kcs1, paf1, pat1, rpb9, snt309, srb2, swi3, tsr2, yap3*	Transcription	
*bem1, bem4, bud27, cwh36, fzo1, hoc1, mid2, nup120, nup133, rmd7, sac6, smi1, ssd1, tpm1, yil090w*	Biogenesis of cellular components	
*meh1, trp1, vac14, vma3, vma10, vma12, vma16, vma21*	Vacuolar function	
*cds1, csg2, erg28, idp1, tco89, trp4*	Metabolism	
*arg82, bck1, fab1, ras2, slt2*	Signal transduction	
*asc1, rpl13b, rps6a*	Protein synthesis	
*kti12, slg1*	Cell rescue, defense, and virulence	
*ybl006c, ydr008c, ydr149c, ydr433w, yel044w, ygr196c, yhr167w, ykl037w, ykl118w, ylr315w, ylr322w, ylr331c, ylr368w, yml095c-a, ymr003w, ynl080c, ynl133c, yor258w*	Unknown function	
*pkc1,ole1,erg6,ssd1,uth1*	Actin polarization/depolarization and the response to cell wall stress. Cell membrane composition/cell wall stability	(van Voorst et al., 2006)
*bem2*	Rho GTPase activating protein	
*slg1*	Sensor-transducer of the stress-activated PKC1-MPK1 kinase pathway	
*gim4* and *gim5*	Encoding sub-units of the hetero-oligomeric co-chaperone Gim C complex	
*ada3, gcn5, spt3* and *spt7 of the saga*	Histone acetyl transferase	
*fen1, plc1, erg6* and *sur4*	Lipid biosynthesis	
*vps34, vac14* and *fab1*	Phosphatidylinositol 3,5-bisphosphate synthesis	
*tps1*	Trehalose-6-phosphate synthase	
*asr1p*	Transcription factor	
*ura7*	CTP synthase isozyme	(Yazawa et al., 2007)
*gal6*	Cysteine aminopeptidase	
*cyb5*	Cytochrome b5	(Yoshikawa et al., 2009)
*yor139c*	Dubious open reading frame; partially overlaps SFL1	
*hsp26*	Heat shock protein	(Jiménez-Martí et al., 2009)
*rtc3*	Involved in RNA metabolism	
*yhr087w*	Unknown	
*pgk1*	Phosphoglycerate kinase glycolytic	
*fps1*	Plasma membrane aquaglyceroporin	(Bai et al., 2004)
*bdp1*	Essential subunit of RNA polymerase III transcription factor (TFIIIB)	
*csl4*	Subunit of the exosome	
*cwc25*	Component of a complex containing Cef1p	
*hts1*	Cytoplasmic and mitochondrial histidine tRNA synthetase	
*irr1*	Subunit of the cohesion complex	
*med8*	Subunit of the RNA polymerase II	
*mpe1*	Essential conserved subunit of CPF (cleavage and polyadenylation factor	
*prp11*	Subunit of the SF3a splicing factor	
*rrp3*	mRNA splicing factor	
*spp381*	One of six subunits of the RNA polymerase III transcription initiation factor complex (TFIIIC)	
*tfc1*	required for rRNA processing	
*fhl1*		
*etp1*	Protein of unknown function required for growth on ethanol	(Snowdon et al., 2009)
*hsp104*	Heat shock protein	(Stanley et al., 2010)
*hsp12*	Heat shock protein	
*ctt1*	Cytosolic catalase T	
*ddr2*	Unknown	
*tdh1*	Glyceraldehyde‐3‐phosphate dehydrogenase	
*tsl1*	Large subunit of trehalose 6‐phosphate synthase	
*tps1*	Synthase subunit of trehalose‐6‐phosphate synthase	
*ald4*	Mitochondrial aldehyde dehydrogenase	
*glk1*	Glucokinase	
*ygp1*	Cell wall	
*pyc1*	Pyruvate carboxylase isoform	
*dak1*	Dihydroxyacetone kinase	
*hxk1*	Hexokinase isoenzyme	
*pgk1*	3‐phosphoglycerate kinase	
*spi1*	Glycosylphosphatidylinositol	
*cyc7*	Cytochrome c isoform 7	
*yer053c, ydr516c, ybr139w*	Unknown	
*swe1*	negative regulator of mitosis	
*spt3*	Subunit of the SAGA (Spt-Ada-Gcn5-acetyltransferase)	
	encodes Na^+^-ATPase	
*ede1*	Endocytic protein	(Lewis et al., 2010)
*elo1*	Elongase I (fatty acid elongation)	
*tps1*	Trehalose-6-phosphate synthase	
*msn2*	Transcriptional activator	
*gpb2*	PKA signaling	
*dog1*	2-deoxyglucose-6-phosphate phosphatase	(Hong et al., 2010)
*hal1*	Cytoplasmic protein involved in halotolerance	
*ino*1	Inositol-3-phosphate synthase	
*ole1*	Δ9 fatty acid desaturase	(Kim et al., 2011)
*rcn1*	Regulator of calcineurin	(Anderson et al., 2011)
*rsa3*	Ribosome maturation	
*ydr307w,yhl042w*	Unknown	(Kasavi et al., 2014)
		
*asr1p*	Ring/PHD finger protein, it has a role in organization of septins and the actin cytoskeleton	(Zou et al., 2015)
*hst4*	Histone deacetylase	(Jimenez and Benitez, 1988)
*vrs70*	Vacuolar protein sorting	
*ybl059w*	Unknown function	
*pca1*	P-type cation	
*hem13*	Hem biosynthesis	
*prt1*	elF3 subunit	
*mex67*	Component of nuclear pore	
*sec24*	Vesicle formation in Endoplasmic reticulum to Golgi transport	(Riles and Fay, 2019) (Sandberg et al., 2019) (Takahashi et al., 2001, Pais et al., 2013) (Hirasawa et al., 2007, Baerends et al., 2009, Swinnen et al., 2015)
*elm1* *PMA1, TRK1* *ath1* *pat1, ade1, kin3* *tat1, ap3, lys2, leu2, his3, met15, tat2*	Encoding serine/threonine protein kinase Electrochemical gradient Trehalose DNA damage repair and chromosome integrity Amino acids and nutrient uptake	

It has been demonstrated that various yeast strains exhibit distinct abilities to grow or survive in the presence of ethanol (Davis López et al., 2018). For instance, *S. cerevisiae* strains vary in their ability to tolerate ethanol, with tolerances ranging from 7% to 14% (v/v) ethanol. This implies that some strains carry specific alleles that allow for better tolerance to the toxic effects of ethanol (Mukherjee et al., 2014). However, it is challenging to measure the universal and absolute levels of ethanol tolerance predominantly because of the lack of a widely accepted method for defining ethanol tolerance.

Adaptive laboratory evolution (ALE) is a well-established method for studying molecular evolution and adaptive changes that accumulate in microbial populations through long-term selection under specified growth conditions (Dragosits and Mattanovich, 2013, Morard et al., 2019). In a successful ALE experiment, multiple lineages of mutated strains with improved fitness can be obtained, and then the genetic determinants resolved by sequencing and reverse engineering (Fletcher et al., 2017). Therefore, ALE has been used as a powerful complementary technique besides metabolic engineering to optimize cellular fitness (Chatterjee and Yuan, 2006). The most common objectives for ALE include optimization of growth on a specific nutrient (e.g. xylose), tolerating physical and chemical stresses (e.g. high temperature or biomass hydrolysate inhibitors), and increasing the titer of novel substances through product-growth coupling metabolic designs (Caspeta et al., 2014, Zhu et al., 2018). Ethanol toxicity is one of the most common bottlenecks in biofuel production using *S. cerevisiae*. ALE has been previously used in various studies to identify biological pathways involved in ethanol toxicity in yeast (Teixeira et al., 2009, Sandberg et al., 2019). For example, Jimenez et al. (1988) applied ALE to develop a yeast isolate in media containing 8% v/v ethanol and observed adaptation through alterations in cell wall composition (Alper et al., 2006). In another study, the ALE experiment was performed on the haploid laboratory strain *S. cerevisiae* W303-1A in continuous mode for 192 days and, which resulted in 486 generations with a feed containing 8.5% v/v ethanol. These evolved strains were able to tolerate higher ethanol concentrations up to 9% v/v (Stanley et al., 2010). In an ALE experiment using isogenic yeast populations with different initial ploidy in a continuous turbidostat with a stepwise increase in ethanol level up to 12% v/v, mutations were found in genes involved in cell cycle regulation, respiration, and DNA repair mechanisms (Jimenez and Benitez, 1988). Furthermore, by applying a protein–protein interaction (PPI) network approach, Kasavi et al. (2014) identified two previously uncharacterized genes, YDR307W and YHL042W, that were associated with improved tolerance to ethanol (Voordeckers et al., 2015). During ALE, multiple mutations are generated; however, typically only a few determine the desired change in the phenotype. These causal mutations are identified via reverse engineering (Della-Bianca et al., 2013, Kasavi et al., 2014). In this study, we aimed to identify genetic mutations that confer tolerance to high levels of ethanol (up to 11%) and investigate whether they also lead to improved ethanol production phenotype. We chose a haploid laboratory strain of *S. cerevisiae*, CEN.PK113-7D, to ease the genotype-phenotype correlation.

## Materials and methods

### Strains and media

The laboratory haploid strain *S. cerevisiae* CEN.PK113-7D (*MATa URA3 HIS3 LEU2 TRP1 MAL2-8c SUC2*) was used as the parental strain for ALE. For reverse engineering, *S. cerevisiae* CEN.PK113-5D (*MATa Δura3 HIS3 LEU2 TRP1 MAL2-8c SUC2*) was transformed with the episomal vector for expression of Cas9 protein with a kanamycin resistance marker (strain ST8251, Table 1S) (Turanlı-Yıldız et al., 2017). To maintain the selection for Cas9, G418 (Sigma–Aldrich) was supplemented into media at 200 mg/L. All *S. cerevisiae* strains used in this study (Table 1S) were derived from the CEN.PK strain family background. The yeast strain constructs were designed for CRISPR-assisted marker-based integration (Milne et al., 2020). The standard lithium acetate method was used for yeast transformation (Wang et al., 2020). The correct yeast transformants were identified by amplifying ∼1 kb of the genomic region containing the expected mutation by a set of primers listed in Table 2S, and followed by Sanger sequencing (Eurofins, Germany).

For yeast transformants, synthetic complete medium without uracil (SC-URA) containing 20 g/L glucose, 6.7 g/L yeast nitrogen base, and drop-out medium supplements without uracil was used. For ALE experiment, yeast peptone dextrose broth (YPD) and molasses medium were used. The same batch of molasses was used for all experiments.The YPD included 10 g/L yeast extract, 20 g/L peptone and 20 g/L glucose, unless otherwise stated. The molasses medium for ALE experiments was prepared by adding 1 g/L ammonium sulfate and 1 g/L urea to sugarcane molasses (20 Brix).The pH of this medium was adjusted to 4.5 by sulfuric acid (De Oliveira Lino et al., 2018). Prior to medium preparation, the molasses was centrifuged (5000 × *g* for 25 min, at 4°C) to remove the solid impurities and autoclaved at 121°C for 10 min. The ammonium sulfate and urea were filter sterilized (0.22 µm).

For ethanol production assays, the cells were cultivated in 24 deep-well plates containing YPD medium inoculated with overnight pre-culture to get an optical density (OD_600_) of 0.1. Then the yeast cells were inoculated into the YPD medium to initiate the fermentation and incubated at 30°C with shaking at 250 rpm in the aerobic phase. After eight hours of growth at 30°C, glucose was added to the final concentration of 220 g/L to mimic the sequential-batch fermentation, and the plates were moved to an anaerobic chamber flushed with N_2_ gas to ensure anaerobic condition. The chamber was then incubated at 30°C with shaking at 70 rpm for ethanol production. The ethanol yield was defined as the mass ratio of the ethanol titer over consumed glucose (Chang et al., 2018). All the experiments were run in triplicates, with mean and standard deviation values reported.

### ALE experiments

**Figure 1.**
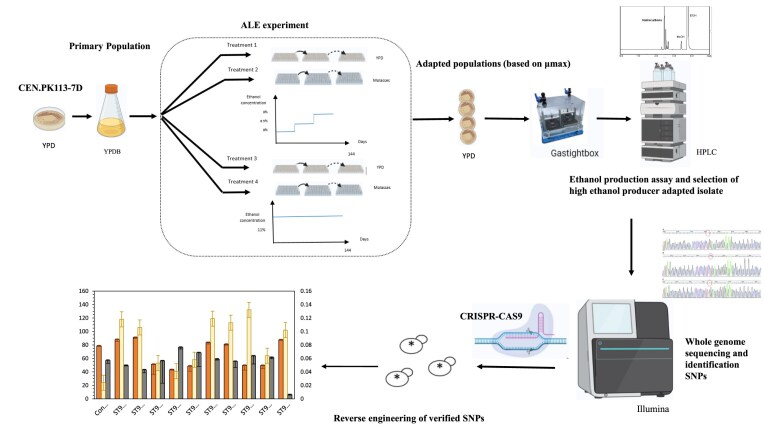
ALE of *S. cerevisiae* CEN.PK113-7D. The evolved clones after serial batch culture and selection for ethanol tolerance were sequenced by whole-genome sequencing to identify the underlying variants responsible for the adaptation. The selected variants were validated using CRISPR-Cas9 and the ethanol production and ethanol tolerance of the engineered CEN.PK113-7D cells. (Treatment 1: increasing ethanol stress in YPD, Treatment 2: increasing ethanol stress in molasses, treatment 3: constant ethanol stress in YPD, Treatment 4: constant ethanol stress in molasses.

The ALE experiment to evolve yeast cells with elevated tolerance to ethanol was carried out by sequential batch cultivations using haploid laboratory *S. cerevisiae* strain CEN.PK113-7D. A single colony of the strain CEN.PK113-7D was used to inoculate 20 mL YPD medium in a 100 mL shake flask. This so-called primary population was grown overnight until the middle of the exponential growth phase and then was inoculated to fresh YPD medium in 96-well microplates at 30°C and 70 rpm. To start ALE experiments, an adequate amount of the cells was then transferred into a fresh medium to get initial OD_600_ of 0.1. A schematic overview of the experimental design is shown in Fig. 1. In total, four different treatments were carried out with either YPD or molasses as the media, and with increasing ethanol concentration of 8% to 9% v/v or with a constant amount of ethanol concentration of 11% v/v (Fig. 1). For each treatment, five biological replicates were included, which resulted in 20 independent evolutionary lines.

In treatments 1 and 2 (Fig. 1), the strains were transferred into fresh medium every 3 days, and the ethanol concentration was increased when a stable growth rate was observed for the previous lower ethanol content over at least 8 transfers. For treatments 3 and 4, the transfer of the isolates into fresh medium was done every week, as the high ethanol concentration of 11% hindered significantly the cell growth. For all the treatments, the ALE experiment was run for a total duration of 144 days. The microplates were sealed with the Breathe-easy^TM^ membrane, followed by shaking at 30°C and 70 rpm using microplate shaker TITRAMAX1000 (Heidolph Instruments, Germany). The outermost wells of the 96-well plate, i.e. the first and 8^th^ rows, as well as the first and 12^th^ columns were not used in order to eliminate the effect of medium evaporation.

To calculate the specific growth rate for each cultivation, an exponential growth function was used. Since the inoculation volume was similar across all the tested wells in a given experiment, the starting OD_600_ values were assumed to be identical for all wells. These values were then chosen as the baseline for calculating the lag time. The average specific growth rate was calculated using the following equation:


(1) \begin{eqnarray*}
\mu = \left(\text{lnX}_{2} - \text{lnX}_{1} \right)/\left( \mathrm{t}_{2} - \mathrm{t}_{1} \right),
\end{eqnarray*}

where X_1_ and X_2_ are the cell densit ies at time t_1_ and t_2_, respectively (Walker-Caprioglio et al., 1985, Horinouchi et al., 2014). µ^max^ was determined directly from the graphical representation of the µ values obtained from growing the yeast in different ethanol concentrations. After each passage, 0.2 mL of culture was added to 0.2 mL of a 40% (v/v) glycerol solution as a backup of the lines, and the samples were stored at −80°C (Ghiaci et al., 2013).

### Whole-genome sequencing

Whole-genome sequencing was performed on the parental strain, sarving as the ancestral reference for the ALE experiments, as well as the evolved clones. This approach enabled the establishment of a precise baseline genotype for our specific parental lineage, allowing for robust variant analysis.The evolved clones were selected from different population samples by streaking glycerol stocks from the corresponding population samples on YPD plates. Three swabs from each clone (named E1–E4) were subsequently grown in YPD liquid medium. Total chromosomal DNA from parental strain, E1–E4 clones were extracted for sequencing after inoculation from glycerol stock and overnight culture in 2.5 mL of YPD in a 14 mL culture tube. The DNA extraction was performed using the Qiagen genomic tip Kit and the final DNA concentrations were measured using Qubit (Invitrogen Qubit 2.0 Fluorometer). The purified DNA samples were subjected to sequencing using NextSeq 500 (Illumina), with the NextSeq Mid Output v2 Kit (300 Cycles). The paired-end reads (2×150 bp) with 100-fold genomic coverage of the *S. cerevisiae* genome size (12 Mb) were chosen. Above 80% of the reads from all lanes were passed the quality score of Q30. Trimmomatic (Bolger et al. 2014) for trimming the adaptor sequences (default parameters) from the pair-end sequencing data in all samples was used. Each sample resulted in over 10 million mappable reads, providing an average mapped sequence coverage of above 250x for all samples. The qualified reads from 12 samples were mapped to the CEN.PK113-7D reference genome (NCBI BioProject: PRJNA5295, Nijkamp et al., 2012) using BWA software (Heng and Richard, 2009). A comprehensive filtering pipeline was then applied to identify and remove pre-existing single nucleotide polymorphisms (SNPs) and structural variants (SVs) present in our parental strain. This refined parental genome sequence was subsequently used as the primary reference for mapping the evolved clones, enabling accurate identification of *de novo* mutations that emerged during the adaptive evolution process. The GATK was used following its Best Practices recommendations (Schmidt, 2009) for variant calling choosing for base quality score recalibration, indel realignment, and duplicate removal across all 12 samples (DePristo et al., 2011, Van der Auwera et al., 2013). For sorting and indexing of the alignment files, SAMtools was used (Li et al., 2009).

### DNA elements and plasmids

All polymerase chain reactions (PCR) were done with Phusion Hot Start II High-Fidelity PCR Master Mix (Thermo Scientific, USA), using the manufacturer’s instructions. The linear fragments containing the 5'-adaptor, the sgRNA, the repair fragment for the introduction of the specific mutations, and the 3'-adaptor were ordered from GeneArt (Thermo Scientific, USA) (Table 3S). These fragments were individually fused with the linear pMEL10 backbone, which was amplified with overhangs containing the 5'-adaptor and the 3'-adaptor.

*Escherichia coli* DH5α was used for all plasmid cloning and propagation experiments (Lee and Kim, 2020). *Escherichia coli* strains were grown in lysogeny broth (LB) medium and supplemented with 100 mg/L ampicillin when required. The plasmids containing both guide RNA cassettes and the donor fragments for mutation introduction were constructed using the Gibson assembly (New England BioLabs). All the purified plasmids were then sent for sequencing with SNR52 as the sequencing primer. Plasmids pCfB9345-pCfB9352 were generated (Eurofins Genomics, Germany) and confirmed by Sanger sequencing (Table 4S).

The plasmids pCfB9345 to pCfB9352 were individually transformed into ST8251 using the LiAc/SS carrier DNA/PEG method (Entian and Kotter, 2007). All the mutations were generated via the CRISPR-Cas9 method, using URA3 plasmid harboring both a guide RNA cassette and the donor fragment for mutation introduction.

### Analytical methods

To quantify the concentrations of ethanol and glucose, samples were analyzed with high-performance liquid chromatography (HPLC) (Ultimate 3000, Thermo Scientific, Waltham, Massachusetts, USA). The Aminex HPX-87H ion exclusion column (Bio-Rad, Hercules, California, USA) was used as the stationary phase, and 5 mM sulfuric acid was used as the mobile phase. A total volume of 10 μL of each sample was injected into the column operating at 30°C with a mobile-phase flow rate of 0.6 mL/min. The compounds were detected using a refractive index detector. The standards were prepared by diluting stock solutions of glucose and ethanol. Cell growth was estimated through measuring absorbance at 600 nm in 96-well microtiter plates using a BioTek Synergy MX plate reader.

## Results and discussion

### ALE to improve ethanol tolerance

**Figure 2.**
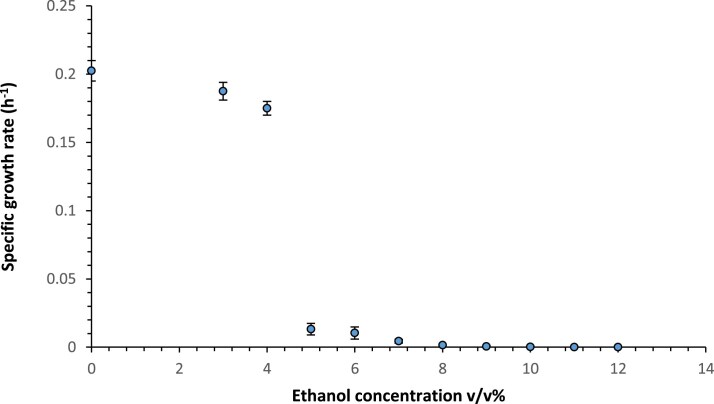
Comparison of maximum specific growth rate of the *S. cerevisiae* CEN.PK113-7D in culture medium without ethanol and media supplemented with different concentrations of ethanol (from 1% to 12% v/v). Data shown are mean values ± SDs of triplicates.

**Figure 3.**
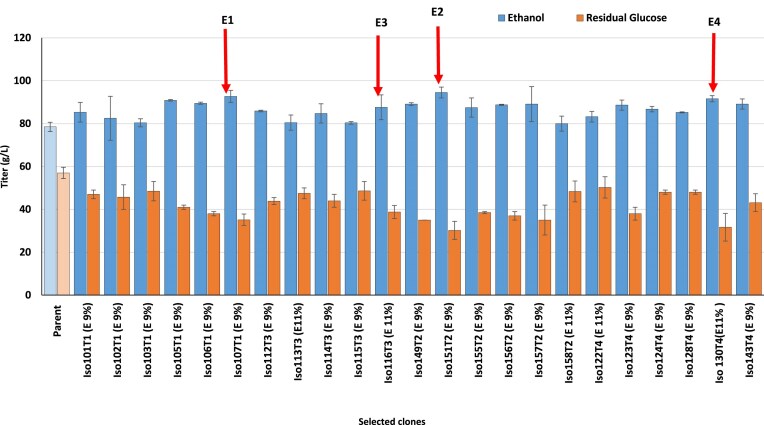
Ethanol titer and residual glucose concentration for the parent strain and the evolved clones isolated from the individual lines and cultured in YPD medium supplemented with 9% (E 9%) or 11% v/v (E 11%) ethanol. The clones with improved ethanol production (E1–E4) were selected and further validated for ethanol production by cultivation in in YPD medium containing 220 g/L glucose, after 36 h fermentation at 30°C and 70 rpm shaking. Data shown are mean values ± SDs of triplicates. Statistical difference between parent and evolved strains was determined by the two-tailed Student’s t test (*P* < 0.05).

**Figure 4.**
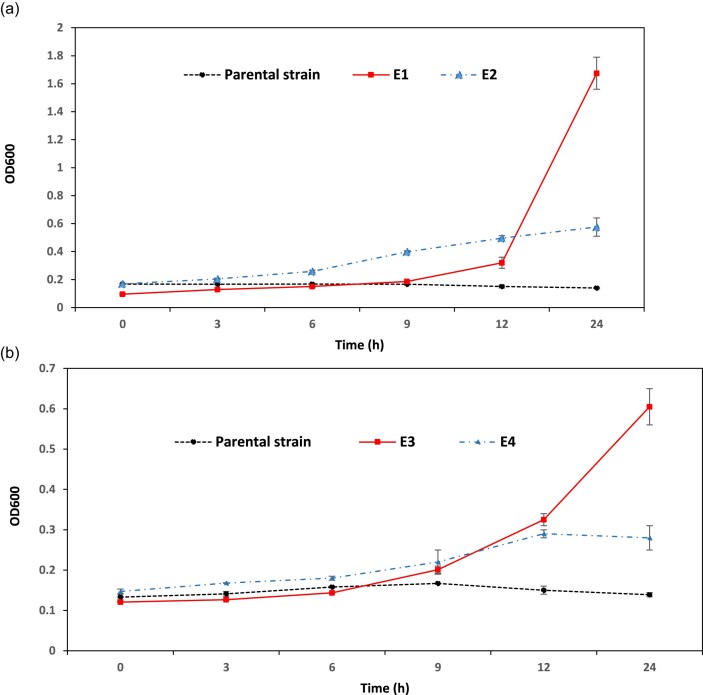
Comparing the growth curve of *S. cerevisiae* CEN.PK113-7D as parent strain with the chosen evolved clones in YPD media in the 250 mL flask supplemented with 9% (v/v) ethanol (4a), and 11% (v/v) ethanol (4b). Three independent sets of cultivation were performed for each clone, and error bars indicate the SDs. Data shown are mean values ± SDs of triplicates.

We aimed to investigate the improvement of ethanol tolerance and its production in *S. cerevisiae* CEN.PK113-7D via ALE. To determine the starting point for ALE experiments, at first, the inhibitory concentrations of ethanol on the growth of the strain CEN.PK113-7D was assessed (Fig. 2). Based on this experiment, the growth was barely detectable at ethanol concentration of 8% v/v. For the ALE conditions, nutrient-rich YPD and industrially-relevant molasses media were used. Ethanol concentration was either gradually increased from 8% v/v to 9% v/v (treatments 1 and 2, Table 2) or maintained at 11% v/v (treatments 3 and 4, Table 2). Each of these four treatments was carried out in with five biological replicates, resulting in five independent evolution lines for each treatment (a total of 20 evolved cell lines). The maximum specific growth rates of the evolved cells were monitored over the period of evolution and the evolved cell lines were selected based on improvements in maximum specific growth rates. Four or five clones from the best-evolved cell lines, in terms of maximum specific growth rate improvement, were selected for further cultivation in YPD liquid medium for ethanol assay (Fig. 3). Eventually, four ethanol-tolerant evolved clones showing the highest ethanol titers (E1–E4) were chosen (Table 2). Clones E1 and E2 were selected from YPD and molasses media, respectively, with ethanol concentrations gradually increased from 8% to 9% v/v. Whereas clones E3 and E4 were chosen from YPD and molasses media, respectively, at a constant 11% v/v ethanol. The selected clones were re-evaluated for their growth performance under the ethanol stress conditions in which they were originally evolved. To enable a more accurate assessment of growth characteristics, cultivations were scaled up from microplate assays to 250 mL shake flasks. Growth profiles were subsequently compared with those of the parental *S. cerevisiae* CEN.PK113-7D strain. This revealed that despite their shared parental lineage, each evolved clone exhibited distinct growth dynamics following ALE (Fig. 4).The highest adaptive gain we obtained was for the evolved clone E1 in YPD with increasing ethanol concentration from 8 to 9% v/v, while the highest ethanol production titer was observed for the clone E2 that was evolved under the same ethanol stressor concentration but in molasses (Table 2). Also, the evolved clone E4 in molasses with constant ethanol concentration of 11% showed higher titers of ethanol compared to the similar evolutionary treatments but with YPD as the medium. Given that the ethanol titer produced by parental strain under similar condition was 78.5 ± 2.68 g/L, we observed an increase in the production capabilities for all the selected evolved clones listed in Table 2. All four evolved clones consumed more glucose than the parental strain, and the residual glucose after fermentation by evolved clones was reduced by ∼30% compared to the parental strain. Clones E2 and E4 exhibited the highest glucose consumption, respectively. The ethanol yield on glucose improved by 8.1% for E1 clone, and by 4.8%–7.5% for the other clones.

**Table 2.** Characteristics of selected evolved clones.

Treatment number	Specifications	# of evolution line	Selected clone	Initial µ_max_ (h^−1^)	Final µ_max_ (h^−1^)	Adaptive gain	EtOH titer (g/L)*	Residual glucose (g/L)	EtOH yield (g EtOH/g Glucose)
1	Evolved in YPD with increasing EtOH concentration from 8% to 9% v/v	5	(Iso107T1) **E1**	0.0240	0.1150	0.0910	92.69 ± 2.80	35.14 ± 2.67	0.5014 ± 0.01
2	Evolved in molasses with increasing EtOH concentration from 8% to 9% v/v	5	(Iso151T2) **E2**	0.0240	0.0690	0.0450	94.50 ± 2.52	30.18 ± 4.22	0.4978 ± 0.02
3	Evolved in YPD with a constant EtOH concentration of 11% v/v	5	(Iso116T3) **E3**	0.0002	0.0530	0.0528	87.63 ± 5.76	38.76 ± 3.05	0.4834 ± 0.01
4	Evolved in molasses with a constant EtOH concentration of 11% v/v	5	(Iso130T4) **E4**	0.0002	0.0281	0.0278	91.59 ± 1.44	31.64 ± 6.44	0.4862 ± 0.01
–	Parental strain CEN.PK 113–7D	–	–	–	–	–	78.51 ± 0.70	57.01 ± 4.24	0.4615 ± 0.02

* Ethanol production was measured in YPD medium containing 220 g/L glucose, after 36 h incubation at 30°C and 70 rpm.

Adaptive gain: specific growth rate at the end of experiments—specific growth rate at the beginning of experiments.

### Reverse engineering to identify causal mutations

While the parental strain utilized for ALE was designated as CEN.PK113-7D, its genomic profile, as determined by whole-genome sequencing, exhibited minor divergence from the publicly deposited TU Delft reference sequence. This subtle variation is likely attributable to cumulative genetic drift arising from historical distribution and routine laboratory propagation. Our bioinformatics workflow was specifically designed to account for this by employing the de novo sequenced parental strain as the foundational reference for all subsequent mutational analyses of the evolved isolates.

To decipher the causal mutations, the genomes of evolved clones E1–E3 were re-sequenced. A total of 406 single-nucleotide variations (SNPs) within coding regions of the genes, including 82 missense mutations, 324 silent mutations, 32 duplications, and 23 deletions from clones E1–E3 were found. The data obtained from whole-genome sequencing of E4 could not be analyzed due to the very large number of identified mutations. The identified SNPs were also validated using Sanger sequencing of the genomic DNA of clones using the primers listed in Table 2S. Furthermore, some overlapping mutations together with similar SNPs in four genes in different clones were identified. These four overlapping missense SNPs included *ANR2*  ^A495T^, *RGA1*  ^P621G^, *RGA2*  ^K114I^, *LPX1*  ^R107K^, and which were observed in clones E1–E3. The *RKI1*  ^I208V^ and the *LRE*  ^S317G^*and CYC2*  ^N342A^ were also detected in E1 and E2. In the *ANR2* gene, three SNPs were found in all three evolved clones, with two of them being identical. The indels were identified mostly in non-coding regions and were excluded from the validation. The majority of the mutations were observed in the clones evolved in YPD media (Table 5S). Surprisingly, identical missense SNPs were identified in *ANR2, RGA1, RGA2, LPX1* across all three independently evolved strains exposed to 9% or 11% v/v ethanol. This strongly suggests a selective advantage under ethanol stress and the contribution of these mutations to the observed improvements in growth rates and ethanol production compared to the parental strain, as shown in Figs. 3 and 4.

**Figure 5.**
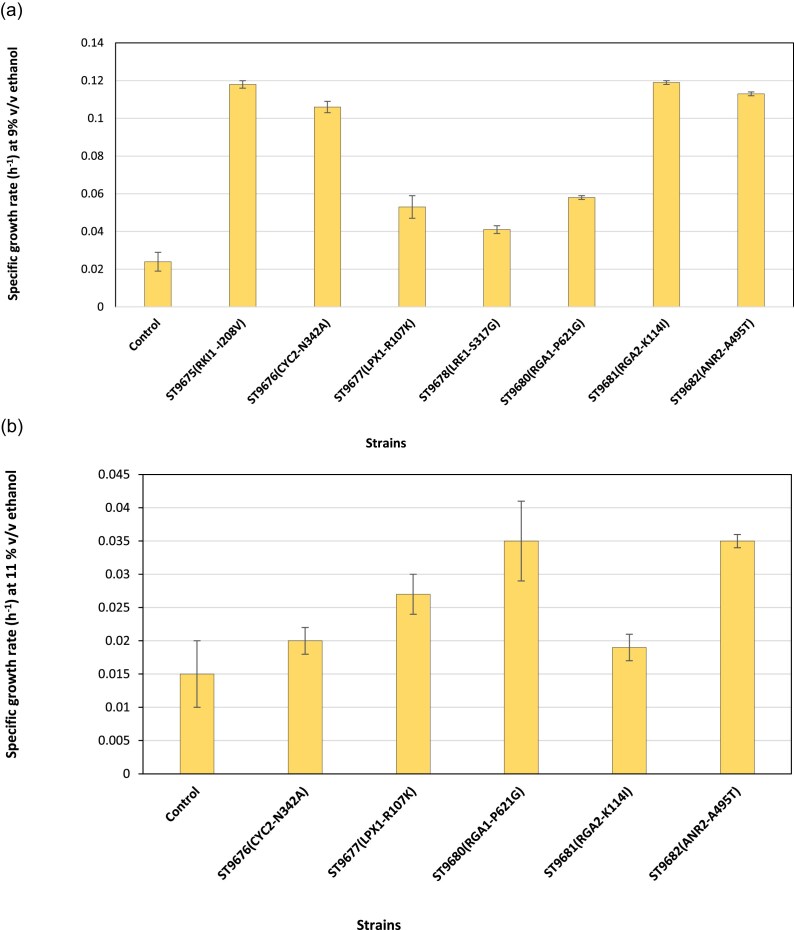
Specific growth rates of the engineered strains carrying stress-induced SNPs were cultured in YPD medium containing 9% (a) or 11% (b) v/v ethanol compared to the parental strain. Each mutant was tested under the same ethanol concentration from which the corresponding SNP was originally identified. Strains harboring individual SNPs exhibited higher maximum specific growth rates than the parental control strain. Statistical difference between the control and the strains was determined by a two-tailed Student’s t-test (*P* < 0.05).

The mutated genes are involved in multiple processes including stress response, cell cycle, membrane and cell wall composition/structure, sugar metabolism, ribosome biogenesis, lipid metabolism, and RNA processing in mitochondria (Table 5S). To investigate the impact of the identified mutations, we reverse-engineered the SNPs that were common for all three evolved strains, *ANR2*  ^A495T^, *RGA1*  ^P621G^, *RGA2*  ^K114I^, *LPX1*  ^R107K^, *RKI1*  ^I208V^, *LRE*  ^S317G^, and *CYC2*  ^N342A^ (Table 6S). The resulting reverse engineered strains were tested for growth in YPD media supplemented with 9% v/v or 11% v/v ethanol (Fig. 5a and b, respectively).

**Figure 6.**
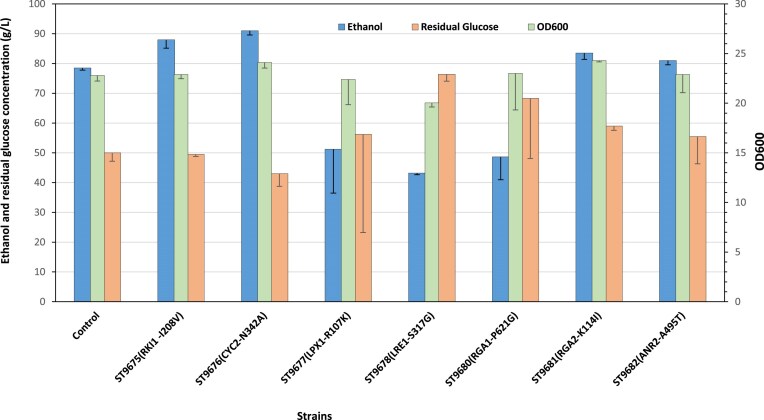
Ethanol production and residual glucose concentrations of engineered strains carrying SNPs. Ethanol production was measured in YPD medium containing 220 g/L glucose, after 36 h fermentation at 30°C and 70 rpm shaking. Four replicate sets of cultivations were performed for each strain and error bars indicate the SDs.

All the engineered strains demonstrated a higher maximum specific growth rate compared to the parental strain. The highest improvement on 9% v/v ethanol was for *RKI1*  ^I208V^ mutation, while at 11% for *RGA1*  ^P621G^ mutation (Fig. 5b). Subsequently, we tested the ethanol production by the reverse-engineered strains on YPD medium to initiate the fermentation and incubated at 30°C with shaking at 250 rpm in the aerobic phase. After 8 h of growth at 30°C, glucose was added to the final concentration of 220 g/L to mimic the sequential-batch fermentation, and the plates were moved to an anaerobic chamber flushed with N_2_ gas to ensure anaerobic condition. The chamber was subsequently incubated at 30°C with shaking at 70 rpm for ethanol production. Four of the mutations, i.e. *RKI1*  ^I208V^*, CYC2*  ^N342A^, *RGA2*  ^K114I^, and *ANR2*  ^A495T^ significantly improved the ethanol production (Fig. 6). The maximum ethanol titers reached 91.0 ± 1.4 g/L in ST9676 (*CYC2*  ^N342A^) and 88 ± 2.8 g/L in ST9675 (*RKI1*  ^I208V^), while the parental strain produced 78.5 ± 0.7 g/L (Fig. 6). Although ethanol tolerance was improved by introducing either of the selected mutations, some mutations resulted in significantly lower ethanol production. For instance, ST9677 (*LPX1*^R107K^), ST9680 (*RGA1*^P621G^), and ST9678 (*LRE*  ^S317G^) produced 51.2 ± 14.1 g/L, 48.7 ± 7.6 g/L, and 43.2 ± 0.5 g/L ethanol, respectively (Fig. 6).

**Figure 7.**
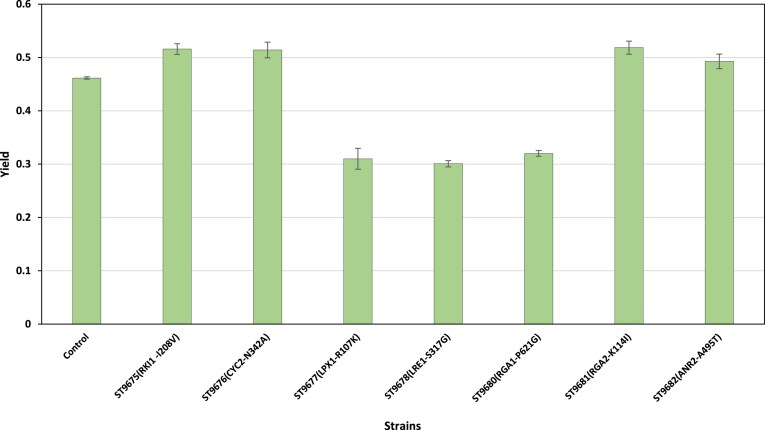
Ethanol yields values of engineered strains harboring SNPs compared to the control strain. Fermentations were conducted in YPD medium containing 220 g/L glucose for 36 h at 30°C with agitation at 70 rpm. Data shown are mean values ± SDs of triplicate. Statistical difference between the control and the strains was determined by a two-tailed Student’s t-test (*P* < 0.05).

The results also demonstrated that the mutant strains ST9675 (RKI1 ^I208V^), ST9676 (CYC2 ^N342A^), and ST9682 (ANR2 ^A495T^) consumed more glucose than the parental strain, as evidenced by significantly lower residual sugar concentrations of 49.5 ± 0.70 g/L, 43.1 ± 4.43 g/L, and 55.5 ± 9.12 g/L, respectively, compared to 57.0 ± 4.24 g/L in the parental strain (*P* < 0.05). Ethanol yield and titer were measured to assess potential growth defects associated with the mutations, as well as the ethanol production efficiency of the mutant strains. ST9681 exhibited the highest ethanol yield of 0.518 ± 0.01 g/g among the strains evaluated after 36 h of fermentation. Although ST9676 achieved a higher ethanol titer than ST9681, it exhibited a slightly lower yield of 0.514 ± 0.01 g/g and demonstrated a superior growth rate. Furthermore, ST9675 recorded an ethanol yield of 0.515 g/g with the highest ethanol tolerance, while the yield of the parental strain was 0.46 g/g. This improvement suggests that the mutant strains have enhanced metabolic pathways for more efficient conversion of glucose to ethanol. (Fig. 7).

To our knowledge, none of these genes investigated in this study have been previously reported in ethanol tolerance, nor have these mutations been found in a laboratory or industrial strains so far.

The *CYC2* gene encodes a protein involved in the import of cytochrome c into mitochondria (Graille et al., 2005, Chen et al., 2012). Cyc2p is an outer-membrane protein, fused cytochrome-porin that transfers an electron into the electron transport chain to produce ATP (Bernard et al., 2005). The FAD molecule binds non-covalently to the C-domain of the Cyc2p terminal, causing the oxidation and reduction function of the protein in the heme lyase reaction (Bonnard et al., 2010, Chen et al., 2014). The results demonstrated a higher threshold for ethanol stress due to point mutations in the *CYC2*. Maintaining the cellular redox balance is essential for living cells to sustain metabolism. Therefore, such a self-balance in cofactor systems can maintain functional stability and dynamic homeostasis in a given redox state automatically, allowing cells to adapt to the ethanol stress.

The Rki1p (Ribose 5-phosphate isomerase) can accelerate the formation of glycolysis pathway precursors, such as glyceraldehyde 3-phosphate and fructose 6-phosphate, which ultimately leads to increased fermentation rates (Bai et al., 2008, Yau et al., 2013). The dynamic rerouting of the metabolic flux to the pentose phosphate pathway, with the concomitant generation of the reduced electron carrier nicotinamide adenine dinucleotide phosphate (NADPH), is a conserved post-translational response to stress. Moreover, isomerase activity of Rki1p regulates the production of ribulose-5-phosphate, leading to the conversion of redox cofactors of the transhydrogenase type (NADP^+^ + NADH →NADPH + NAD^+^) and the conversion of excess NADH to NAD^+^. This ultimately causes reduced glycerol production and enhanced ethanol yield (Cadière et al., 2011, Van Aalst et al., 2022).

Prior studies have proven that plasma membrane and cell wall stability are key factors in ethanol tolerance (Semkiv et al., 2014). ANR2p is a protein with unknown function, but Crurrie et al. (2014) identified it as one of the lipid droplet (LD) proteins. LDs are omnipresent dynamic organelles that store and supply lipids in all eukaryotic cells for energy metabolism, membrane synthesis, and production of essential lipids (Avrahami-Moyal et al., 2012). On the other hand, there is a hypothesis that yeast cells increase unsaturated lipid content to deter interior digestion and maintain an optimal membrane thickness as ethanol concentration increases during anaerobic fermentation. Changes in plasma membrane composition provide an important survival factor for yeast cells to prevent ethanol toxicity (Currie et al., 2014). Prior studies changes indicate that in the plasma membrane composition of yeast cells in response to ethanol toxicity enhance membrane fluidity to compensate for ethanol-induced disruption, and mutations in the *ANR2* could be effective in regulating lipid synthesis (Dinh et al., 2008, Pol et al., 2014).

Rho-type GTPase is involved in a variety of functions in the yeast life cycle. The Rho subfamily of the Ras superfamily of GTPases acts as a molecular shift and modulates many cellular processes. An adequate activity of GTPase is a basis for yeast cells to maintain their intracellular physiological pH, because H^+^ produced during ethanolic fermentation needs to be continuously pumped out of the yeast cells by the proton motive force, driven by GTPase or ATPase (Kim et al., 2011). Therefore, the ethanol inhibition in the fermentation is expected to be reduced by properly neutralizing its environmental H^+^. If the H^+^ gradient is decreases across the membranes when due to ATPase is inhibition by the high ethanol concentration, the driving force to pump H^+^ out of the cells will be insufficient.

Lre1p regulated trehalose levels, resistance to heat stress, cell separation, and cellular polarity by inhibiting the protein kinase Cbk1. The protein is involved in the control of cell wall structure and stress response (Sopko et al., 2007).

The *LPX1*-encoded protein plays a vital role in acyl hydrolysis. Esterase, lipase, and phospholipase activities of Lpx1p have important functions in the synthesis of fatty acids and is necessary for lipid storage and lipid mobilization. The protein is also involved in the synthesis of unsaturated fatty acids such as oleic acid (Versele and Thevelein, 2001, Thoms et al., 2008). Interestingly, mutations in genes linked to the pentose phosphate pathway (*RKI1*) and Rho GTPase-activating protein (RGA2) significantly increased their fitness in response to ethanol stress.On the other hand, three of the mutations (*RGA1, LPX1*, and *LRE1*) tested at a 9% v/v ethanol concentration did not significantly increase fitness, although they were observed at a high frequency in our adapted clones. This is indicative of achievable epistatic interactions with other mutations (Jimenez and Benitez, 1988, Semkiv et al., 2014).

In the future, these mutations can be transferred into industrial ethanol-producing strains, such as Ethanol Red®, to test whether ethanol production can be improved.

## Conclusion

We identified several new mutations that increase ethanol tolerance in the yeast *S. cerevisiae* through ALE, followed by whole-genome resequencing and reverse engineering. Some of these mutations led to enhanced ethanol production.

While this study elucidated the individual contributions of single mutations to ethanol-related phenotypes, future research should aim to reconstruct combinations of mutations to evaluate their potential synergistic or cumulative effects. Given that multiple mutations in evolved strains may exhibit epistatic interactions, their combined impact cannot be reliably predicted from the effects of individual mutations alone. To further validate the applicability of these findings, engineered combinations will also be tested in industrial yeast strains under relevant fermentation conditions

## Supplementary Material

foaf058_Supplemental_File

## Data Availability

All data generated or analyzed during this study are included in this published article (and its supplementary information files).
