# Supplementary material for: Adaptive laboratory evolution of Saccharomyces cerevisiae CEN.PK 113-7D to enhance ethanol tolerance
Source: FEMS Yeast Res. 2025 Nov 28;25:foaf058. doi: 10.1093/femsyr/foaf058 (PMC12671053; doi:10.1093/femsyr/foaf058)
Supplement: foaf058_Supplemental_File [file foaf058_supplemental_file.docx]

**Supplementary materials**

Fatemeh Sheikhi^1, 2^, Mahsa Babaei^3^, Khosrow Rostami^1*^, Mehrdad Azin^1^, Mohammad Ali Asadollahi^4^, Payam Ghiaci^5^, Mansour Ebrahimi^6^, Amir Feizi^7^, Irina Borodina^3^*

^1^ Department of Biotechnology, Iranian Research Organization for Science and Technology (IROST), Tehran 331319-3685, Iran

^2^ Sugarcane Training and Research Institute, Khuzestan 63451-96316, Iran

^3^ The Novo Nordisk Foundation Center for Biosustainability, Technical University of Denmark, DK-2800 Kgs. Lyngby, Denmark

^4^ Department of Biotechnology, Faculty of Biological Science and Technology, University of Isfahan, Isfahan 81746-73441, Iran

^5^ Department of Bioreﬁnery and Energy, High-throughput Centre, Research Institutes of Sweden, Örnsköldsvik 89250,

^6^ SwedenBioinformatics Research Group, Green Research Center, University of Qom, Iran

^7^ OMass Therapeutics, Oxford, England

*****Corresponding Authors:

Irina Borodina

The Novo Nordisk Foundation Center for Biosustainability, Technical University of Denmark, Kemitorvet Building 220, 2800 Kongens Lyngby, Denmark.

Email: irbo@biosustain.dtu.dk

Khosrow Rostami

Department of Biotechnology, Iranian Research Organization for Science and Technology (IROST), Tehran, Iran. Email: [rostami@irost.ir](mailto:rostami@irost.ir)

Table 1S: Strains used or generated in this work.

| Strain ID | Genotype | Parental strain | Introduced plasmid | Reference |
| --- | --- | --- | --- | --- |
| *S. cerevisiae* CEN.PK113-7D | MATa *URA3 TRP1 LEU2 HIS3* |  |  | Nijkamp et al.,2012 |
| S. cerevisiae CEN.PK113-5D | MATa *ura3Δ* |  |  | YME lab at DTU Biosustain |
| ST8251 | MATa ura3Δ cas9 | CEN.PK113-5D | pCfB2312 | Milne et al., 2020 |
| ST9675 | MATa *URA3* RKI1 (I208V) | ST8251 | pCfB9345 | This work |
| ST9676 | MATa *URA3* CYC2 (N342A) | ST8251 | pCfB9346 | This work |
| ST9677 | MATa *URA3* LPX1(R107K) | ST8251 | pCfB9347 | This work |
| ST9678 | MATa *URA3* LRE1 (S317G) | ST8251 | pCfB9348 | This work |
| ST9680 | MATa *URA3* RGA1(P621G) | ST8251 | pCfB9350 | This work |
| ST9681 | MATa *URA3* RGA2(K114I) | ST8251 | pCfB9351 | This work |
| ST9682 | MATa *URA3* ANR2(A495T) | ST8251 | pCfB9352 | This work |

Table 2S: Primers used in this study

| Primer ID | Sequence (5'→3') | Description |
| --- | --- | --- |
| 26177 | CCGTGTGTTGTATGGGTA | Colony PCR primers to amplify region  in CMR2 gene containing the mutation |
| 26178 | CTG TGA ATT CGT GCA ACT T |  |
| 26179 | CAGCTCTCAATGGGTCA | Colony PCR primers to amplify region  in LRE1gene containing the mutation |
| 26180 | ATC ATC GCT AAT ACA CCA GC |  |
| 26181 | GCCGAACGAAGATAATGAA | Colony PCR primers to amplify region  in UBP2 gene containing the mutation |
| 26182 | TGG AGG GAC GTA TTC AGT A |  |
| 26183 | TGGTACCGGAATAGTTACTG | Colony PCR primers to amplify region  in CYC2 gene containing the mutation |
| 26184 | GTT TAT ACA CAT TAT CGG AGT TC |  |
| 26185 | GGT TCA AAG AGT TAC TAC ACG T | Colony PCR primers to amplify region  in ANR2 gene containing the mutation |
| 26186 | TAA CAG AGA TTT CCC AAA CAT |  |
| 26187 | GACAAACAAATCTAGCCTGA | Colony PCR primers to amplify region  in VHS3 gene containing the mutation |
| 26188 | TTG GTT GCT GAG TTA GTT AG |  |
| 26189 | GCCGCTTCTCACATCAT | Colony PCR primers to amplify region  in PGM2 gene containing the mutation |
| 26190 | CGT ACT TTG CCC AGA ATT |  |
| 26191 | GGT ACT AGG ATT GGT ATG ACA | Colony PCR primers to amplify region  in CMR2 gene containing the mutation |
| 26192 | CCA GAT CAC CAG TTC TCA A |  |
| 26193 | GCTGATGAAGTGGATGAGA | Colony PCR primers to amplify region  in RKI1 gene containing the mutation |
| 26194 | GGT AAC TTC AAC ACT ACC GT |  |
| 26195 | GGAACACGCAGACTGTA | Colony PCR primers to amplify region  in TUB3 gene containing the mutation |
| 26196 | AGA ACT CCT CAG CGT AAG A |  |
| 26197 | AGCAGACCCATCCAACAT | Colony PCR primers to amplify region  in MTH1 gene containing the mutation |
| 26198 | GGT AAA CTT GTG CCT GAC A |  |
| 26199 | TAGATGATTCAGCTCTCAATGG | Colony PCR primers to amplify region  in LRT1 gene containing the mutation |
| 26200 | GTT GAC ACC GTT GAC TGA A |  |
| 26201 | GGAGTATGCGCCATATGA | Colony PCR primers to amplify region  in RTK1 gene containing the mutation |
| 26202 | TCT TTA TTC CCG TGT TGC T |  |
| 26203 | GAATGATCCAATCGATAGCT | Colony PCR primers to amplify region  in SHE4 gene containing the mutation |
| 26204 | TCG ACC ACT GCT TTA TCA A |  |
| 26207 | CCGTTCGCATTGCACAA | Colony PCR primers to amplify region  in YAP1801 gene containing the mutation |
| 26208 | GAG GTT GTT AGC ATA TTG GT |  |
| 26209 | GAAGTTCGTGACAGCTAGT | Colony PCR primers to amplify region  in SEC12 gene containing the mutation |
| 26210 | CCAATTCACCCTTCATGT |  |
| 26211 | ACAATAGCTCTGCAACA | Colony PCR primers to amplify region  in FAB1 gene containing the mutation |
| 26212 | ATCGCTAGTATATCGGGATTC |  |
| 26213 | ACGCTGTGTGCGACAGA | Colony PCR primers to amplify region  in LPX1 gene containing the mutation |
| 26214 | CGA GAT GGT AGT TCT GTA GAG T |  |
| 26215 | AGC TGA CCC TAT TAA TGA CCA | Colony PCR primers to amplify region  in RGA2 gene containing the mutation |
| 26216 | CAG TGC TAT TTC TGG GTG T |  |
| 26217 | AGATGGCAAAGATCAGTA | Colony PCR primers to amplify region  in IAH1 gene containing the mutation |
| 26218 | CCA TCA TCT AGC ACA TCT CT |  |
| 26267 | CAAGATCATTGTCACGAAGGT | Colony PCR primers to amplify region  in RGA1 gene containing the mutation |
| 26268 | CTG ACA CCT TGT ATA GAT TGC T |  |
| 23260 | AGGTCAGGCGGAATGGCACTTCgatcatttatctttcactgcggagaag | Primers to amplify pMEL10 backbone from pQC003. |
| 23261 | AGGCTAGGTGGAGGCTCAGTGattcgccctatagtgagtcgtattac |  |
| SNR52 | TAGATTTTGTAGTGCCCTCT | Sequencing primer to sequence fragment mentioned above |

Table 3S. Synthetic DNA fragment sequences

| Name | Sequence |
| --- | --- |
| RKI-dis | AATTGCATGCTGAAAAAGTTGACATCAGACAAGGAGGTTCTGCTAAAGCAGGTCCTGTTGTAACTGACAACAACAATTTTATAGTCGACGCTGATTTCGGTGAAATTTCCGATCCAAGAAAATTGCATAGAGAAATCAAACTGTTAGTGGGCGTGGTGGA |
| CYC2-dis | TATTATGCCTGTGTTGGCATTGGTATGCGGCCCCGAAAGTTATATTAGTAGCATATCTGGAAGAAAATACGCTCTGAACCAAGGCCCCGTCGGAGGGTTACTTTCCAAAGAAGGCTGGAACTCCGATAATGTGTATAAACTTTCATAATTTAACGTTCGA |
| LPX1-  dis | GCCGCTCTCGCACTGCCACTAGGCTTAACCTTGTGTTTTTGCACGGCAGCGGCATGAGTAAGGTGGTATGGCAGTATTACTTACCACGCCTAGTAGCCGCCGATGCGGAGGGCAATTATGCCATCGACAAGGTTTTGTTGATCGACCAGGTTAACCACGG |
| LRE1-  dis | CAGGCAATGACACCTTCTTCCCTGGCCTATACCCCTTCTAAACTAGCATCTACTCCCGCAACACCAGTCTCATTCTACGATGGCAACGCAGACATTAACTTAGAAAGTGATAATTTTCCACTAAAAGATAACCCTAGATATGCCAAGGATGGTTATCC |
| RGA1-  dis | GAAACGATCCCTCGCTTGATAAAGAAATTGTCACGGCAGAGCACTATCTGAAGCAATTAAAAATAAATTTAAAGGGACTCGAGTCGCAGAGAGAAGAATTAATGAAAGAGATCACTGAAATGAAGTCTATGAAAGAGGCTCTACGCCGACATATTGAATC |
| RGA2-  dis | CTATTGTTCCAATTGTTTCAGATGCTGTCGGTGCAGCAACCGTATAAAGAACTTAAAGTATGCAAAGACGATACGCGGCCTCTGCTGCATGGATTGTCACGAGAAATTGTTGAGAAAAAAACAACTATTACTGGAAAATCAAACAAAAAATTCATCGA |
| ANR2-  dis | ATTACTATCGCAGCTTCATTTTTAACCAAGTTAAATTTGGATTGCTTTAGCAAACAAGATCATCAATTCGTAAAAGACATAACCCTCAAGTGGTTCCAAAGAAGCATTGATATTAGCAACCTACCAGAATGTTTGGGAAATCTCTGTTAACCACTAGAAA |

Table 4S: Plasmids used in this study

| Plasmid ID | Feature | Parental  plasmid | DNA  insert | Description | Construction approaches /reference |
| --- | --- | --- | --- | --- | --- |
| pQC003 | 2μ ori, KlURA3,Amp, GAL80,gRNA | NA | NA | plasmid, used as template  for backbone preparation for the  plasmid via PCR, for the  mutation inrtoduction | Chalmers  University |
| pCfB9345 | 2μ ori, KlURA3,Amp,RKI 1, gRNA | pQC003 | RKI1-dis | Point mutation introduction into PKI1 | Gibsson assembly |
| pCfB9346 | 2μ ori, KlURA3,Amp,CYC2, gRNA | pQC004 | CYC2-dis | Point mutation introduction into CYC2 | Gibsson assembly |
| pCfB9347 | 2μ ori, KlURA3,Amp,LPX1, gRNA | pQC005 | LPX1-dis | Point mutation introduction into LPX1 | Gibsson assembly |
| pCfB9348 | 2μ ori, KlURA3,Amp,LRE1, gRNA | pQC006 | LRE1-dis | Point mutation introduction into LER1 | Gibsson assembly |
| pCfB9350 | 2μ ori, KlURA3,Amp,RGA1, Grna | pQC007 | RGA1-dis | Point mutation introduction into RGA1 | Gibsson assembly |
| pCfB9351 | 2μ ori, KlURA3,Amp,RGA2, gRNA | pQC008 | RGA2-dis | Point mutation introduction into RGA2 | Gibsson assembly |
| pCfB9352 | 2μ ori, KlURA3,Amp,ANR1, gRNA | pQC009 | ANR1-dis | Point mutation introduction into ANR1 | Gibsson assembly |

Table 5S: The lines used for each treatment (ALE) and the adaptive gain obtained for each of them.

| Treatment no | Specifications | lines | Initial µ_max_ | Final µ_max_ | Adaptive gain |
| --- | --- | --- | --- | --- | --- |
| 1 | YPD with increasing EtOH from 8 to 9% v/v | Evolved line1 | 0.0240h^-1^ | 0.115 h^-1^ | 0.0910 |
|  |  | Evolved line2 |  | 0. 0.0395h^-1^ | 0.0155 |
|  |  | Evolved line3 |  | 0.0310h^-1^ | 0.0070 |
|  |  | Evolved line4 |  | 0.0470h^-1^ | 0.0230 |
|  |  | Evolved line5 |  | 0.0390h^-1^ | 0.0150 |
| 2 | Molasses with increasing EtOH from 8 to 9% v/v | Evolved line6 |  | 0.0420h^-1^ | 0.0180 |
|  |  | Evolved line7 |  | 0.0281h^-1^ | 0.0041 |
|  |  | Evolved line8 |  | 0.0690h^-1^ | 0.0450 |
|  |  | Evolved line9 |  | 0.0320h^-1^ | 0.0080 |
|  |  | Evolved line10 |  | 0.0490h^-1^ | 0.0250 |
| 3 | YPD with constant 11% v/v EtOH | Evolved line11 | 0.0002h^-1^ | 0.0080h^-1^ | 0.0078 |
|  |  | Evolved line12 |  | 0.0530h^-1^ | 0.0528 |
|  |  | Evolved line13 |  | 0.0002 | 0.0 |
|  |  | Evolved line14 |  | 0.0002 | 0.0 |
|  |  | Evolved line15 |  | 0.0002 | 0.0 |
| 4 | Molasses with constant 11% v/v EtOH | Evolved line16 |  | 0.0002 | 0.0 |
|  |  | Evolved line17 |  | 0.0002 | 0.0 |
|  |  | Evolved line18 |  | 0.0002 | 0.0 |
|  |  | Evolved line19 |  | 0.0280h^-1^ | 0.0278 |
|  |  | Evolved line20 |  | 0.0002 | 0.0 |

Table 6S: The SNPs obtained from WGS analysis along with the function of their corresponding genes.

| **Gene** | **Evolved clone** | **Amino acid change** | **Description** |
| --- | --- | --- | --- |
|  |  |  |  |
| YOR084W | E1 | Arg107Lys | Peroxisomal matrix-localized lipase |
| YOR084W | E3 | Glu69Gln | Peroxisomal matrix-localized lipase |
| PET127 | E1 | Ser549Asn | Protein with a role in 5'-end processing of mitochondrial RNAs |
|  |  |  |  |
| PET127 | E3 | Ser549Asn | Protein with a role in 5'-end processing of mitochondrial RNAs |
|  |  |  |  |
| YOR93C | E3 | Ala1277Val | Protein of unknown function |
|  |  |  |  |
| YOR93C | E1 | Ala1277Val |  |
|  |  |  |  |
| YGR067C | E1 | Ile55Val |  |
| YNRO29C | E1 | Gly355Arg |  |
| YFRO20W | E2 | Glu61Asp |  |
| YOR019W | E1 | Lys193Asn |  |
| YOR019W | E2 | Tyr347His |  |
| YOR097C | E2 | Pro154Ser |  |
| YOR062C | E1 | Val203Ala |  |
| YDL022C | E1 | Gln14* |  |
| YDL022C | E2 | Gln14* |  |
| YOR024W | E1 | *92Tyr |  |
| YOR024W | E3 | *92Tyr |  |
| YOR062C | E3 | Tyr23Phe |  |
| YOR062C | E3 | His32Asn |  |
| YFR020W | E1 | Glu61Asp |  |
| YNR029C | E3 | Gly355Arg |  |
| YOR062C | E3 | Tyr264* |  |
| YOR062C | E1 | Asp94Asn |  |
| YDL301W | E1 | Pro907Arg | Dead Box Protein |
| YOR046C | E3 | Ala451Thr | Dead Box Protein(DBP5) |
| PRT5 | E1 | Gln407Lys | High-copy Mep Suppressor |
| YOR086C | E1 | Thr16Ala | Lipid-binding ER protein |
|  |  |  |  |
| YOR057W | E3 | Pro153Leu | Suppressor of G2 (Two) allele of skp1 |
|  |  |  |  |
| YOR127W | E3 | Glu621Gly | Rho GTPase Activating Protein |
|  |  |  |  |

| **Gene** | **Evolved clone** | **Amino acid change** | **Description** |
| --- | --- | --- | --- |
| YOR076C | E1 | Thr353Arg | GTP-binding protein that couples the Ski complex and exosome |
|  |  |  |  |
| YOR076C | E1 | Pro98Ser | GTP-binding protein that couples the Ski complex and exosome |
|  |  |  |  |
| YOR076C | E3 | Pro98Ser | GTP-binding protein that couples the Ski complex and exosome |
|  |  |  |  |
| GAT2 | E1 | Ser90Ile | Protein containing GATA family zinc finger motifs |
|  |  |  |  |
| GAT2 | E3 | Ser90Ile | Protein containing GATA family zinc finger motifs |
| STD1 | E1 | Pro146Leu | Protein involved in control of glucose-regulated gene expression |
| YDR277C | E1 | Cys329Trp | Negative regulator of the glucose-sensing signal transduction pathway |
| BUD21 | E1 | Ser127Asn | Component of small ribosomal subunit (SSU) processosome |
| YOR123C | E1 | Arg310Ile | Component of the Paf1 complex; which associates with RNA polymerase II and is involved in histone methylation |
| YOR080W | E3 | Ile472Met | Origin-binding F-box protein,DIA2 |
| YOR017W | E3 | Met671Val | CSS2 |
| YOR117W | E1 | Gln407Lys | ATPase of the 19S Regulatory particle of the 26S proteasome(PRT5) |
| YOR124C | E3 | Trp90Lys | Ubiqitin-specific protease |
| YOR035C | E1 | Lys25Asn | Protein containing a UCS (UNC-45/CRO1/SHE4) domain |
| YOR026W | E3 | Ala100Ser | Kinetochore |
| YOR075W | E1 | Asn191Lys | UFE1 |
| YMR136W | E1 | Ser90Ile | Protein containing GATA family zinc finger |
| YML124C | E2 | Ala388Val | Alpha-tubulin |
| YOR073W | E1 | Thr230Ser | SGO1 |
| YDL025C | E1 | Pro585Thr | Ribosome biogenesis |
| YDL025W | E1 | Arg15Ser | Ribosome biogenesis |
| YOR032C | E3 | Ser280Cys | HMS1 |
| YDL202W | E3 | Arg107Lys | Mitochondrial ribosomal protein of the large subunit |
| YOR043W | E3 | Ser242Leu | WHI1 |
|  |  |  |  |

| **Gene** | **Evolved clone** | **Amino acid change** | **Description** |
| --- | --- | --- | --- |
| YOR047C | E1 | Pro146Leu | Protein involved in control of glucose-regulated gene expression,STD1 |
|  |  |  |  |
| YOR045W | E3 | Ala11Thr | Translocase of the Outer Mitochondrial membrane |
|  |  |  |  |
| ANR | E1 | Ser464Pro | Protein of unknown function; may have a role in lipid metabolism |
| ANR | E3 | Ser464Pro | Protein of unknown function; may have a role in lipid metabolism |
| ANR | E3 | Ala495Thr | Protein of unknown function; may have a role in lipid metabolism |
| ANR | E2 | Ala495Thr | Protein of unknown function; may have a role in lipid metabolism |
| RGA2 | E3 | Lys114Ile | GTPase-activating protein for polarity |
| RGA2 | E2 | Lys114Ile | GTPase-activating protein for polarity |
| RGA2 | E1 | Lys114Ile | GTPase-activating protein for polarity |
| CYC2 | E3 | Asp342Ala | Cytochrome oxidase 2 |
| LER | E3 | Ser317Gly | Protein involved in control of cell wall structure and stress response |
| RSB1 | E1 | Gly16Ser | putative sphinghoied long chain base(LCB) efflux transpoter |
| RKI1 | E1 | Ilu208Val | Ribose 5- phosphate ketol-Isomerase |
| RGA1 | E1 | Pro621Gln | Rho GTPase Activating Protein |
| RGA1 | E2 | Pro621Gln | Rho GTPase Activating Protein |
| RGA1 | E3 | Pro621Gln | Rho GTPase Activating Protein |
| LPX1 | E1 | Arg107Lys | Peroxisomal matrix-localized lipase |
| LPX1 | E2 | Arg107Lys | Peroxisomal matrix-localized lipase |
| LPX1 | E3 | Arg107Lys | Peroxisomal matrix-localized lipase |


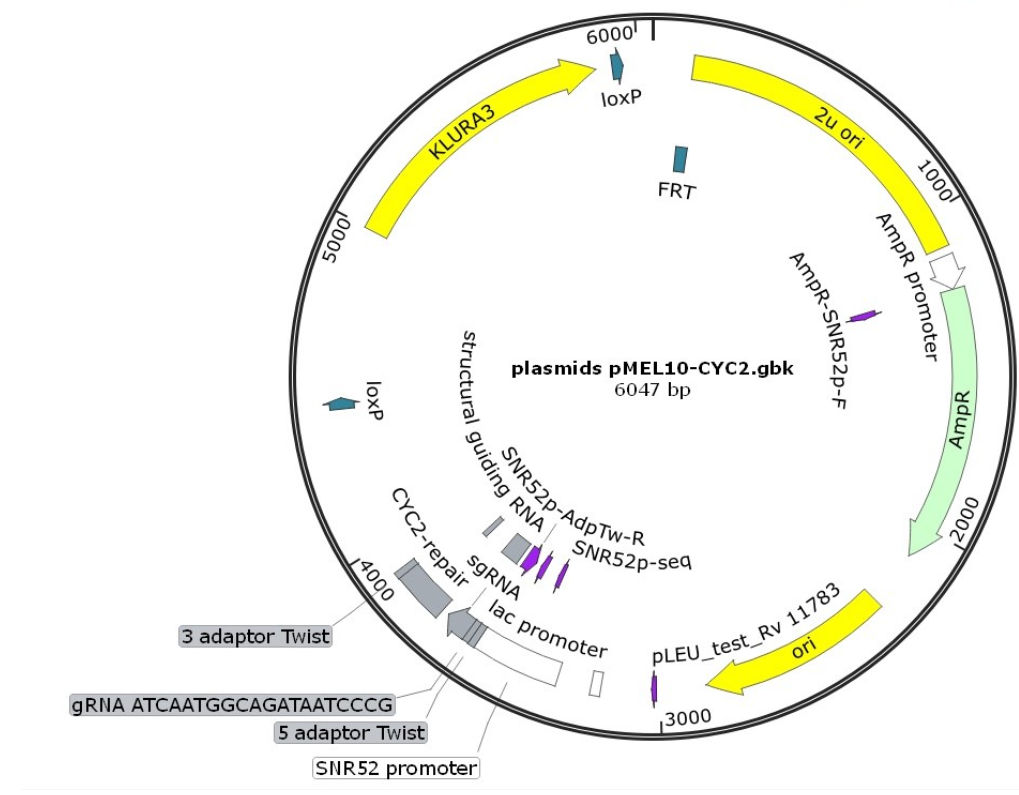


Figure 1S: Plasmid map of pMEL10 harboring gRNA and repair fragment for a generic gene.
